# Machine Learning SNP Based Prediction for Precision Medicine

**DOI:** 10.3389/fgene.2019.00267

**Published:** 2019-03-27

**Authors:** Daniel Sik Wai Ho, William Schierding, Melissa Wake, Richard Saffery, Justin O’Sullivan

**Affiliations:** ^1^Liggins Institute, University of Auckland, Auckland, New Zealand; ^2^Murdoch Children Research Institute, Melbourne, VIC, Australia

**Keywords:** machine learning, polygenic risk score, precision medicine, genetic disease risk prediction, personalized medicine, complex disease risk

## Abstract

In the past decade, precision genomics based medicine has emerged to provide tailored and effective healthcare for patients depending upon their genetic features. Genome Wide Association Studies have also identified population based risk genetic variants for common and complex diseases. In order to meet the full promise of precision medicine, research is attempting to leverage our increasing genomic understanding and further develop personalized medical healthcare through ever more accurate disease risk prediction models. Polygenic risk scoring and machine learning are two primary approaches for disease risk prediction. Despite recent improvements, the results of polygenic risk scoring remain limited due to the approaches that are currently used. By contrast, machine learning algorithms have increased predictive abilities for complex disease risk. This increase in predictive abilities results from the ability of machine learning algorithms to handle multi-dimensional data. Here, we provide an overview of polygenic risk scoring and machine learning in complex disease risk prediction. We highlight recent machine learning application developments and describe how machine learning approaches can lead to improved complex disease prediction, which will help to incorporate genetic features into future personalized healthcare. Finally, we discuss how the future application of machine learning prediction models might help manage complex disease by providing tissue-specific targets for customized, preventive interventions.

## Precision Medicine

Since the completion of the Human Genome Project, DNA sequencing technologies have been advancing rapidly (Laksman and Detsky, 2011; Johnson, 2017). These advances have been most notable in terms of a dramatic decrease in the cost per base pair sequenced (Schuster, 2008). This has led to an exponential increase in the abundance of individual-specific genotype data and other forms of human biological “omics” information (Laksman and Detsky, 2011; Spiegel and Hawkins, 2012). As a result of these technological developments, the concept of precision medicine, or personalized medicine, has undergone a world-wide upsurge in support as a way of transforming disease prediction, prognosis, and individual participation in preventative strategies (Laksman and Detsky, 2011; Johnson, 2017).

The objective of precision medicine is to deliver tailored medical treatments for patients according to their genetic characteristics. This primarily involves customizing proactive and preventive care to maximize medical efficacy and cost-effectiveness (Laksman and Detsky, 2011). Personalization is achieved by integrating and utilizing various types of omics information to generate and understand disease risks (Laksman and Detsky, 2011; Spiegel and Hawkins, 2012; Redekop and Mladsi, 2013). The application of precision medicine to pharmacogenomics has allowed for customized drug and dosage use with considerable success. For example, genetic information is regularly incorporated into treatment strategies for trastuzumab treatment for HER2-positive breast cancers, erlotinib for EGFR-overexpressing lung cancers, or imatinib for Philadelphia chromosome-positive chronic myelogenous leukaemias (Salari et al., 2012; Wald and Morris, 2012). However, in the context of population health, it is hotly debated whether precision genomics is yet at a point where it offers cost-benefits over and above fully implemented standard public health approaches.

## Genome-Wide Association Studies

There are millions of single nucleotide polymorphisms (SNPs, also known as genetic variants) in each human genome (Auton et al., 2015). Genome-wide association (GWA) studies identify SNPs that mark genomic regions that are strongly associated with phenotypes in a population (Visscher et al., 2012). These genomic regions must contain the variant that is causally associated with the phenotype, however it does not follow that the SNP that is identified by the GWA study is causal. Notably, many common and complex diseases [e.g., type 2 diabetes (T2D) and obesity] are influenced by multiple SNPs, each with small per-SNP effect sizes (Visscher et al., 2017). Of note, the majority of these SNPs are located in non-coding regions and thus must be indirectly involved in their disease association, likely through tissue-specific regulatory activities (Visscher et al., 2017; Schierding et al., 2018). New methods to understand these regulatory activities include the integration of spatial and temporal aspects of gene expression data (Schierding and O’Sullivan, 2015; Schierding et al., 2016; Fadason et al., 2017, 2018; Nyaga et al., 2018). These approaches are providing insights into the impacts of genetic variants that can reassign population based risk to individualized risk.

## Predicting Risk Scores and AUC

Traditional epidemiology based models of disease risk (with limited predictive power) have been primarily informed by lifestyle risk factors such as family history (Jostins and Barrett, 2011; Wang et al., 2016). Recently, the inclusion of genetic risk factors, including disease or phenotype associated SNPs, into risk modeling has improved the accuracy of individual disease prediction (Jostins and Barrett, 2011; Wang et al., 2016). Perhaps the greatest promise of risk prediction models lies in their potential to guide diease prevention and treatment without the need for costly and potentially adverse medical screening procedures (e.g., invasive biopsies) (Wray et al., 2007; Ashley et al., 2010; Manolio, 2013; Abraham and Inouye, 2015).

Currently, the main focus of developing genetic risk models is to achieve accurate predictive power for recognizing at-risk individuals in a robust manner (Ashley et al., 2010; Manolio, 2013; Montañez et al., 2015). As stated earlier, GWA studies define SNPs according to their association with a disease/phenotype at a population level. Therefore, the incorporation of SNPs into a risk prediction model requires integration into models that score an individual’s genotype to enable the estimation of risk. Genetic risk prediction models are typically constructed by: (1) Polygenic risk scoring; or (2) Machine learning (Wei et al., 2009; Abraham and Inouye, 2015). The predictive performance of both model types is evaluated by receiver operating characteristic curves (ROCs) (Kooperberg et al., 2010; Jostins and Barrett, 2011; Vihinen, 2013; Wang et al., 2016), where the sensitivity and specificity of the predictions are ranked at various cut-off values (Kooperberg et al., 2010; Jostins and Barrett, 2011; Vihinen, 2012; Wang et al., 2016). The area under a ROC curve (AUC) is the probability of the examined model correctly identifying a case out of a randomly chosen pair of case and control samples (Kooperberg et al., 2010; Jostins and Barrett, 2011; Kruppa et al., 2012; Vihinen, 2012; Wang et al., 2016). AUC results range from 0.5 (i.e., random) to 1 (i.e., 100 percent accuracy) (Kooperberg et al., 2010; Jostins and Barrett, 2011; Vihinen, 2012; Wang et al., 2016).

## Polygenic Risk Scoring

Polygenic risk scoring uses a fixed model approach to sum the contribution of a set of risk alleles to a specific complex disease (Belsky et al., 2013; Che and Motsinger-Reif, 2013; Wang et al., 2016; So et al., 2017). Polygenic risk scores can be unweighted or weighted. In weighted polygenic risk scores, the contributions of the risk alleles is typically weighted by their odds ratios or effect sizes (Evans et al., 2009; Purcell et al., 2009; Wei et al., 2009; Carayol et al., 2010; Medicine and Manolio, 2013). By contrast, unweighted polygenic risk scores are equal to the sum of the number of associated variant alleles in a genome. The unweighted model assumes that all variants have an equivalent effect size (Carayol et al., 2010; Abraham and Inouye, 2015; Hettige et al., 2016). This simplistic assumption limits the utility of unweighted polygenic risk scores for complex traits with underlying genetic architectures that include uneven variant effects (Carayol et al., 2010; Abraham and Inouye, 2015; Hettige et al., 2016).

There are two stages to the development of a polygenic risk score: (1) the discovery stage; and (2) the validation stage. The discovery stage of a weighted polygenic risk score uses statistical association testing (e.g., linear or logistic regression) to estimate effect sizes from a large case and control dataset of individual genotype profiles (Evans et al., 2009; Che and Motsinger-Reif, 2013; Dudbridge, 2013). The discovery stage of an unweighted polygenic risk score requires strict SNP selection parameters to prevent incorporation of SNPs with minor effect sizes. In both the weighted and unweighted polygenic risk score, once developed, the discovery model is passed to the validation stage. Validation of the polygenic risk score requires the extraction of informative SNP identities and effect sizes from the discovery set, using a stringent association *p*-value threshold (e.g., 5 × 10^-8^) (Dudbridge, 2013; Wray et al., 2014),which is subsequently passed to a scoring phase of the validation. During this process, the polygenic risk score model is applied to a testing dataset [i.e., an independent set of case and control genotype data (Che and Motsinger-Reif, 2013; Dudbridge, 2013)]. Polygenic risk scores are calculated for each individual genotype profile in the testing data (Che and Motsinger-Reif, 2013; Dudbridge, 2013). The predictive power of the individual polygenic risk scores for the complex trait are then established by the strength of the score associations with the clinically measured outcomes (phenotypes) in the testing dataset (Che and Motsinger-Reif, 2013; Dudbridge, 2013).

Early attempts to use weighted polygenic risk scores, were based on small numbers of highly significant SNPs identified from GWA studies, and achieved only limited predictive value for complex diseases (Amin et al., 2009; Dudbridge, 2013). This illustrates a key limitation of weighted polygenic risk score modeling, specifically the *p*-value threshold for SNP choice in the discovery dataset impacts on the model’s performance and predictive power. The selection of limited numbers of SNPs, with large effect sizes, over-simplifies the biological underpinnings of the complex diseases by ignoring the bulk of the variants that make much smaller individual contributions to the phenotype (Visscher et al., 2017). For example, the average odds ratio per T2D risk allele ranges from 1.02 to 1.35 (Shigemizu et al., 2014). Recent polygenic risk score models incorporate expanded SNP selection to achieve better predictive results for complex polygenic traits (Dudbridge, 2013; Escott-Price et al., 2015; So et al., 2017). For example, the use of relaxed *p*-value thresholds (as high as 0.01, 0.1, and 0.2 etc…) has enabled the development of improved polygenic risk score models for psychiatric diseases, with minimal increases in false positive errors (i.e., the models have an acceptable power-to-noise ratio) (Amin et al., 2009; Kooperberg et al., 2010; Wray et al., 2014). The weighted polygenic risk score approach has enabled the risk prediction of schizophrenia to achieve reasonable efficacy with an AUC of ∼0.65 (Jostins and Barrett, 2011). Similarly, significant results from weighted polygenic risk score predictions were also obtained for other complex traits including Type 1 diabetes and celiac disease (CD) (Jostins and Barrett, 2011; Wray et al., 2014; So et al., 2017).

## Machine Learning Disease Prediction Models

Machine learning approaches adapt a set of sophisticated statistical and computational algorithms (e.g., Support vector machine (SVM) or Random forest) to make predictions by mathematically mapping the complex associations between a set of risk SNPs to complex disease phenotypes (Quinlan, 1990; Wei et al., 2009; Kruppa et al., 2012; Mohri et al., 2012). These methods use supervised or unsupervised approaches to map the associations with complex diseases (Dasgupta et al., 2011). Despite the utility of unsupervised machine learning methods and non-genetic data in disease predictions (Singh and Samavedham, 2015; Worachartcheewan et al., 2015), we will focus the remainder of this manuscript on supervised modeling that is informed by SNP data.

**Figure 1:**
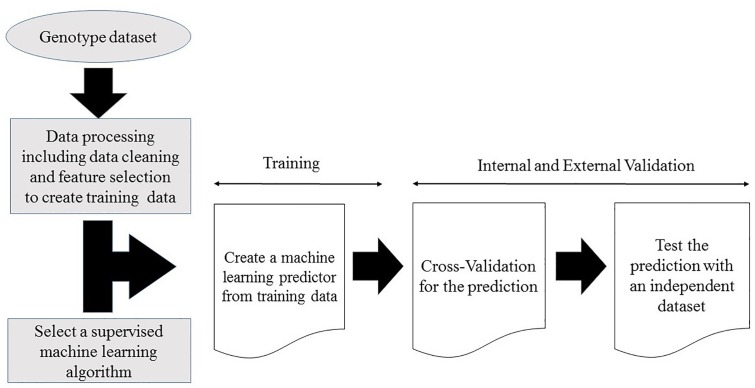
Workflow for creating a supervised machine learning model from a genotype dataset.

Supervised machine learning disease prediction models are generated by training the pre-set learning algorithms to map the relationships between individual sample genotype data and the associated disease (Dasgupta et al., 2011; Okser et al., 2014). Optimal predictive power for the target disease is achieved by mapping the pattern of the selected features (variables) within the training genotype data (Quinlan, 1990; Mohri et al., 2012; Okser et al., 2014). Some models use gradient descent procedures and iterative rounds of parameter estimation to search through the training data space for optimized predictive power (Yuan, 2008; Mehta et al., 2019). This recursive process continues until the optimal predictive performance is reached (Yuan, 2008; Mehta et al., 2019). At the end of the training stage, the models with the maximum predictive power on the training dataset are selected for validation (Vihinen, 2012; Abraham and Inouye, 2015). A generalized workflow for creating a machine learning model from a genotype dataset is illustrated in Figure 1.

During the validation stage, the performance of the predictive machine learning models is evaluated to determine their power for generalized prediction. As with polygenic risk scoring, the validation stage is accomplished by evaluating the algorithm on an independent dataset. The validation stage is essential for ensuring the prediction models do not overfit the training data (Dasgupta et al., 2011; Okser et al., 2014; Abraham and Inouye, 2015). Cross validation is a commonly used procedure for validating the models performance using the original dataset (Schaffer, 1993; Kruppa et al., 2012; Vihinen, 2012; Nguyen et al., 2015; Zhou and Troyanskaya, 2015). However, external validation (testing) using an independent dataset is required to finally confirm the predictive power of a machine learning model. The utility of the algorithm is finally determined through randomized controlled comparisons to current clinical best practice. Only if the algorithm adds information to more accurately stratify populations, predict disease risk or treatment responses does it ultimately prove its clinical utility.

## Factors That Improve the Power of Predictive Models for Complex Diseases

Despite initial promise, the predictive performance of polygenic risk scores for complex diseases has only been moderately successful (Wei et al., 2009; Kruppa et al., 2012; Abraham and Inouye, 2015). A significant contributor to this relatively poor performance revolves about the finding that experimental GWA study data suggests that risk allele contributions to complex diseases have average odds ratios of between 1.1 and 2 (Wray et al., 2007). However, GWA studies are typically underpowered and only capable of detecting risk SNPs with odds ratios of >1.3 (Dudbridge, 2013; Wray et al., 2014). Thus, improving the predictive power of polygenic disease risk models could be as simple as increasing GWA study sample sizes (Wei et al., 2009; Okser et al., 2014; Abraham and Inouye, 2015). Rapidly decreasing DNA sequencing costs have led to meta-GWA studies analyzing datasets containing half a million or more samples (The Wellcome Trust Case Control Consortium, 2007; Amin et al., 2009; Lyall et al., 2018). The use of larger datasets has increased the frequency of detection of SNPs with small effect sizes. Incorporating SNPs with small effect sizes into polygenic risk models has resulted in an increase in the accuracy of complex disease predictions (Wei et al., 2009; Jostins and Barrett, 2011; Vihinen, 2012; Abraham and Inouye, 2015). It remains likely that this trend to use SNPs identified from bigger datasets will continue into the future, with the associated increases in the accuracy of the resulting risk prediction models.

The size of the training and validation datasets is another critical element in machine learning modeling. However, size is not enough and the datasets must be of high quality with accurate phenotyping that ensures the generalizing predictive power of the resultant machine learning models (Vihinen, 2012; Wei et al., 2014). Wei et al. (2013) illustrated the impact of training sample size on the predictive power of a machine learning classification algorithm for inflammatory bowel disease (IBD). The dataset used in the study contained 60,828 individual genotypes from 15 European counties (Wei et al., 2013). A machine learning prediction model for Crohn’s disease (a subtype of IBD) created from a small subset (*n* = 1,327) of the dataset only performed moderately (AUC = 0.6). However, the predictive power of the model improved consistently with increases in size of the training datasets until the predictive performance reached the maximum (AUC = 0.86) with the full training dataset (*n* = 11,943) (Wei et al., 2013).

**Figure 2:**
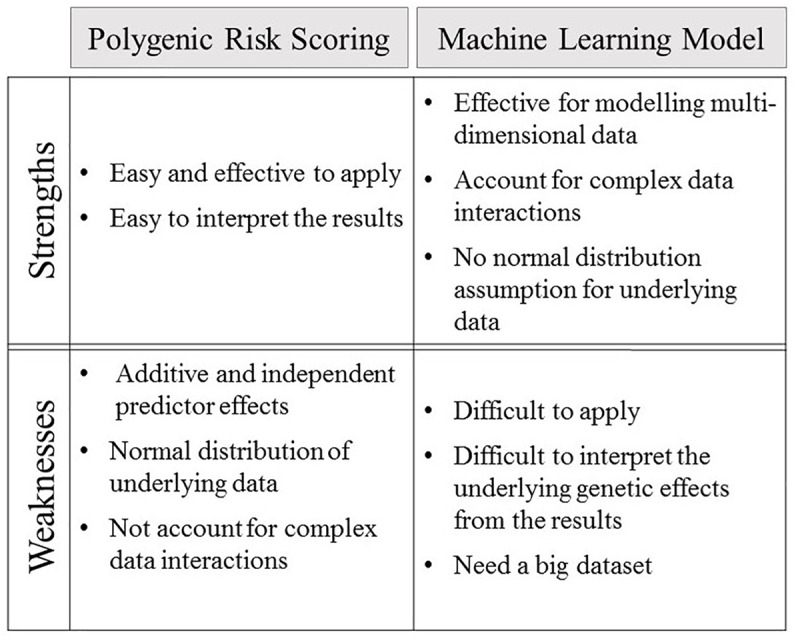
The strengths and weaknesses of polygenic risk scoring and machine learning model.

Technological advances are constantly improving the quality and quantity of the complex integrative datasets that are collected on human phenotypes and disease. Integration of these highly dimensional genomic data within machine learning models can lead to improvements in genetic risk prediction over that achieved for polygenic risk scores (Wei et al., 2009; Okser et al., 2010, 2014; Kruppa et al., 2012; Fourati et al., 2018; Joseph et al., 2018). Polygenic risk score predictions are based on a linear parametric regression model that incorporates strict assumptions, which include additive and independent predictor effects, a normal distribution for the underlying data, and that the data observations are non-correlated (Wei et al., 2009; Abraham et al., 2013; Che and Motsinger-Reif, 2013; Casson and Farmer, 2014; Abraham and Inouye, 2015). These assumptions do not necessarily hold true for the fundamental genetic structures of complex polygenic diseases, thus leading to greatly reduced predictive efficacy (Wei et al., 2009; Abraham et al., 2013; Che and Motsinger-Reif, 2013). Notably, linear additive regression modeling is incapable of accounting for complex interactive effects between associated alleles (Abraham et al., 2013; Che and Motsinger-Reif, 2013; Okser et al., 2014), which have been reported to make major contributions to phenotypes (Furlong, 2013). Thus, linear additive regression based modeling leads polygenic risk scores toward biased and less effective predictions (Clayton, 2009; Huang and Wang, 2012; Che and Motsinger-Reif, 2013; Okser et al., 2014). By contrast, machine learning algorithms employ multivariate, non-parametric methods that robustly recognize patterns from non-normally distributed and strongly correlated data (Wei et al., 2009; Okser et al., 2010, 2014; Ripatti et al., 2010; Silver et al., 2013). The capacity of machine learning algorithms to model highly interactive complex data structures has led to these approaches receiving increasing levels of interest for complex disease prediction (Wei et al., 2009; Okser et al., 2010, 2014; Ripatti et al., 2010; Silver et al., 2013). The strengths and weaknesses of both polygenic risk scoring and predictive machine learning models are summised in Figure 2.

## Machine Learning Feature Selection and Regularization

Data feature selection is the major factor that impacts on a machine learning model’s predictive performance (Okser et al., 2014). Data feature selection occurs during the machine learning training stage with the aim of reducing data dimensionality, removing noisy and irrelevant data, and thus preserving the most useful signals from the dataset (Kwak and Choi, 2002; Okser et al., 2014). Data feature selection procedures can be broadly implemented using filtering, embedded modules, or wrapper methods (Pal and Foody, 2010; Kruppa et al., 2012; Okser et al., 2013, 2014; Shi et al., 2016). The choice of selection procedures depends on the original data attributes and prediction model criteria (Pal and Foody, 2010; Okser et al., 2014). For complex polygenic diseases, SNPs are currently considered the most informative data features within genotype data (Abraham et al., 2013; Okser et al., 2013; Wei et al., 2013; Shi et al., 2016). It is assumed that the SNPs that are selected for inclusion in the predictive models are associated with loci that contribute mechanistically to the underlying disease etiology (Pal and Foody, 2010; Okser et al., 2014; López et al., 2017). Despite this, how the SNP mechanistically contributes to the disease may not be understood. Commonly, in the first stage of the model building, variants within the genotype data are filtered and subdivided into groups according to their GWA study *P*-value thresholds (Wei et al., 2009, 2013; Okser et al., 2013, 2014; Montañez et al., 2015). Embedded methods are implemented inside the model building algorithm and function to select SNPs following the detection of their interactive effects (Okser et al., 2013) and thus enable incorporation of only informative SNPs into the predictors (Wu et al., 2009; Okser et al., 2013; Wei et al., 2013). Wrappers serve the same purpose as embedded methods. However, wrappers are independent stand-alone SNP selection modules implemented before the model building process (Pahikkala et al., 2012; Okser et al., 2013).

Overfitting is a phenomenon whereby models are so closely fitted to a dataset and they cannot be used to generalize to other datasets. The chances of overfitting models can be reduced by regularization, which is a process that maximizes the generalized predictive power of machine learning models (Tibshirani, 1996; Zou and Hastie, 2005; Okser et al., 2014). For example, the two most common types of regression-based regularization are L1 and L2. L1 and L2 regularizations both use a penalized loss function to assign weights that adjust data feature effects and reduce the complexity of the regression models (Tibshirani, 1996; Zou and Hastie, 2005; Okser et al., 2014). L1 regularization sets the weights of non-informative data features to zero, thus eliminating effects and allowing only essential and valuable data feature effects to be included into the machine learning regression modeling (Tibshirani, 1996; Zou and Hastie, 2005; Okser et al., 2014). By contrast, L2 regularization minimizes non-essential data features using non-zero weights (Tibshirani, 1996; Zou and Hastie, 2005; Okser et al., 2014). As a result of this, L2 regularization is not typically used for feature selection.

Regression-based L1-regularization is one of the most commonly used machine learning feature selection methods, with Lasso and Elastic Net currently being the most popular L1 regularization modules (Tibshirani, 1996; Zou and Hastie, 2005; Wu et al., 2009; Okser et al., 2014). There are many examples where L1-regularization has enhanced the machine learning algorithm’s predictive performance for different diseases (Abraham et al., 2013; Wei et al., 2013; Shigemizu et al., 2014; Shieh et al., 2017). For example, Wei et al. (2013) implemented a two-step model training process in the development of an L1-regularized algorithm for Crohn’s disease prediction. Firstly, the Lasso-logistic regression method identified a set of essential and informative SNPs. Subsequently, the selected SNPs were applied to a SVM and a logistic predictor for Crohn’s disease. Following SNP optimization by L1-regularization, both the non-parametric and parametric predictors achieved similar results with an AUC = 0.86 compared to an AUC = 0.73 for the simple polygenic risk score.

Abraham et al. (2014) used six European genotype datasets to develope a Lasso–SVM integrated model, with an AUC = 0.9, for CD. Following data cleaning and adjustment for population structure effects by principal components, Abraham et al. (2014) created a L1-SVM predictor from each dataset with cross-validaion. They then used the other five datasets for external validation. Data feature selection for all the predictors was acomplished by the Lasso method embedded within the SVM algorthm. The best predictor that was generated had an AUC = 0.9 and its clinical utility is being explored for CD prediction (Abraham and Inouye, 2015). Notably, the identification of the essential SNPs by the Lasso-SVM model has provided insights that will help decipher the genetic basis underlying the etiologic pathways of CD pathogenesis.

## Supervised Learning Algorithms

Supervised learning algorithms can be classified as regression-based or tree-based methods (Table 1; Dasgupta et al., 2011; Okser et al., 2014). Logistic regression, linear regression, neural networks, and SVM are popular examples of regression based supervised learning algorithms (Dasgupta et al., 2011; Kruppa et al., 2012). Regression-based supervised learning methods employ polynomial parametric or non-parametric regression methods to map the associations of multidimensional input data to outputs (Dasgupta et al., 2011; Okser et al., 2014; Mehta et al., 2019). By contrast, tree-based supervised learning algorithms, which include Decision trees and Random forests, typically utilize binary decision splitting rule approaches to model the relationships between the input and output data (Dasgupta et al., 2011; Okser et al., 2014; Mehta et al., 2019).

**Table 1:** A brief view of common machine learning algorithms.

Regression based	Examples
Logistic regression	• Use parametric regressions to estimate the probabilities of dichotomous outputs (Dasgupta et al., 2011)	Cox, 1958; Yu et al., 2014; Niriella et al., 2018
Neural Network	• Use multi-layers of non-parametric regressions and transformations to model input data to outputs (Mehta et al., 2019)	Rosenblatt, 1962; Montañez et al., 2015; Xue et al., 2018
Support vector machine (SVM)	• Use non-parametric regressions to model input data for creating multi-dimensional hyperspaces to discriminate the outputs (Yu, 2010)	Corinna and Vladimir, 1995; Abraham et al., 2014; Han, 2018
**Regression based regularization**	
Lasso	• Apply L1 penalized loss functions in regression (Okser et al., 2014)	Tibshirani, 1996; Wei et al., 2013; Song et al., 2018
Elastic net	• Apply L1 and L2 penalized loss functions in regression (Okser et al., 2014)	Zou and Hastie, 2005; Abraham et al., 2013; Rashkin et al., 2018
**Tree-based**	
Decision tree	• Utilize binary decision splitting rule approaches to model the relationships between input data and outputs (Mehta et al., 2019)	Quinlan, 1986; Geurts et al., 2009; Li et al., 2018
Random forest	• Utilize an ensemble of randomized decision trees to model input data to outputs (Mehta et al., 2019)	Breiman, 2001; Worachartcheewan et al., 2015; Dai et al., 2018

*The examples include the founding papers and current examples as at December 2018*.

Regression-based machine learning approaches have been widely employed in risk prediction for many diseases including: cancer; Alzheimer’s; cardiovascular disease; and diabetes (Capriotti et al., 2006; Cruz and Wishart, 2006; Palaniappan and Awang, 2008; Yu, 2010; Zhang and Shen, 2012). For example, an SVM regression-based non-parametric machine learning model of the genetics of type 1 diabetes was built and trained from 3443 individual genotype samples (Mieth et al., 2016) achieving an AUC = 0.84, which is significantly higher than the polygenic risk scoring model AUC = 0.71 (Clayton, 2009; Wei et al., 2009; Jostins and Barrett, 2011). Notably, validation testing confirmed that the predictive power of the non-parametric SVM consistently outperformed the logistic regression control prediction model on two independent datasets (Wei et al., 2009).

Deep learning prediction models developed from neural network algorithms have been gaining a lot of interest following their successful implementation in image recognition and natural language processing applications (He et al., 2016; Young et al., 2018). In genomics, deep learning applications are helping to identify functional DNA sequences, protein binding motifs and epigenetic marks (Alipanahi et al., 2015; Zhou and Troyanskaya, 2015; Zhang et al., 2018). A deep learning model incorporating SNPs associated with obesity has demonstrated a remarkable ability to correctly identify a case out of a randomly chosen pair of case and control samples with an AUC = 0.99 (Montañez et al., 2015). After data cleaning, a genotype dataset of 1997 individuals including 879 cases and 1118 controls with 240,950 SNPs was obtained. The dataset was subsequently filtered into four SNP feature sets, according to *P*-value thresholds obtained from the GWA study. The numbers of SNPs in the feature sets were: 5 (*P*-value: 1 × 10^-5^); 32 (*P*-value: 1 × 10^-4^); 248 (*P*-value: 1 × 10^-3^); and 2465 (*P*-value: 1 × 10^-2^). The feature set with 2465 SNPs (*P*-value: 1 × 10^-2^) was used to construct an artificial neural network (ANN) deep learning model from 60% of the original genotypes as training, 20% as internal validation, and 20% as testing. The ANN deep learning model delivered a significant predictive performance for obesity on the testing set with an AUC = 0.9908 (Montañez et al., 2015). Montañez et al. (2015) clearly demonstrated the ability of the ANN deep leaning algorithm to capture combined SNP effects and predict complex polygenic diseases.

Tree-based machine learning commonly uses a Random Forest algorithm (Jiang et al., 2009; Boulesteix et al., 2012; Touw et al., 2013; López et al., 2017). Random Forest algorithms construct prediction models using an ensemble method with many decision trees. Specifically, Random Forest algorithms select for and evaluate SNPs that are informative in the decision-tree building process (Boulesteix et al., 2012; Nguyen et al., 2015). A strength of Random Forest models is their ability to effectively handle missing and highly dimensional data structures that contain complex interactions (Boulesteix et al., 2012; Nguyen et al., 2015). For example, in a recent study a Random Forest algorithm was used to predict T2D risk, outperforming both SVM, and logistic regression models (López et al., 2017). In this study, a set 1074 individual genotypes and 101 preselected T2D related SNPs were collected and cleaned. The cleaned data (677 samples with 96 related SNPs) were fed into a Random Forest learning algorithm and produced a T2D predictor that delivered an AUC = 0.85 with cross validation (López et al., 2017). In so doing, the Random Forest model also refined the preselected SNPs to identify a subset that are strongly associated with T2D and can be used to interrogate the etiology of the disease (Boulesteix et al., 2012; Nguyen et al., 2015; López et al., 2017). The implementation of Random Forrest is still useful as a machine learning method for complex disease risk modeling (Boulesteix et al., 2012; Chen and Ishwaran, 2012; Austin et al., 2013; López et al., 2017).

## Individual Tissue-Specific Heterogeneity

Although PRS and machine learning approaches have been extensively used in complex disease prediction, little attention has been given to the utility of machine learning applications in calculating tissue-specific disease risk in individuals. This is largely because GWAS studies identify relationships between global somatic SNPs and their associated phenotypes (Visscher et al., 2017). However, GWAS-identified, disease-associated SNPs are recognized as modifying regulatory mechanisms which affect gene expression in a tissue-specific manner (Parker et al., 2013; Ardlie et al., 2015). Therefore, by expanding GWAS methodology to include expression measures (i.e., expression quantitative trait locus, eQTL), genetic analyses could help to interrogate the inter-related biological networks between cell and tissue types that propagate the causal effects to complex diseases (Ardlie et al., 2015; Ongen et al., 2017). For example, incorporating eQTL data led to the identification of adipose-specific gene expression patterns that could have an inferred causal role in obesity (Nica and Dermitzakis, 2013). Similarly, genes with liver specific expression are now thought to be a major contributor to T2D (Rusu et al., 2017). By extending eQTL analyses to include chromatin spatial interaction (Hi-C) data, it was shown that T2D and obesity associated SNPs have spatial-eQTLs which implicate dysfunction of specific regulatory actions in various tissue types (Fadason et al., 2017). These studies strongly suggest that by aggregating biological data types (e.g., DNA, RNA, and epigenetic data), the accumulated result becomes a tissue-specific network analysis of associated dysfunctionally regulated genes. Thus, specific disease risk to individuals should be calculated using a tissue-by-tissue approach, concluding with tissue-specific networks and pathways that are particular to the development of a disease.

In so doing, it may be possible to leverage the tissue-effect heterogeneity of patients by identifying the correct genes and tissue loads to provide essential targets for potential therapeutic interventions leading to enhanced therapeutic effectiveness. The tissue-effect heterogeneity could also help to recognize individual subtypes of complex disease, facilitating personalized treatments. By targeting the causal associated SNP tissue-specific effects, predictions of patient specific tissue-effect disease risks could provide informative biomarkers for early disease prevention, bringing about a substantial reduction of later disease burdens and costs. Zhou and Troyanskaya (2015) have utilized the deep learning algorithm to predict the functional effects of non-coding variants by modeling the pattern of genomic and chromatin profiling information. They have been able to employ this method to distinguish important eQTLs and disease-related SNPs from various eQTL and SNP databases. Nevertheless, despite the immense promise of machine learning, it is important to recognize that at present there is insufficient research in their application for the identification of disease-associated tissue-specific risks. It is likely that these caveats will be attenuated in the near future through advanced tissue-specific studies of complex traits and disease.

## Conclusion

Precision medicine is a rapidly advancing field that already provides customized medical treatments and preventative interventions for specific diseases, especially cancer. Using a patient’s SNPs to predict individual disease risks is an essential element for delivering the fuller promise of precision medicine. Polygenic risk scoring is a straightforward model for assigning genetic risk to individual outcomes, but has achieved only limited success in complex disease predictions due to its dependency on linear regression. The polygenic risk scoring method is ineffective in modeling highly dimensional genotype data with complex interactions. By contrast, the strength of machine learning data modeling in complex disease prediction lies in its handling of interactive high-dimensional data. Coupled with large new population datasets with high-quality phenotyping at different stages in the lifecourse, machine learning models are capable of classifying individual disease risks with high precision. Notably, machine learning predictors that include tissue-specific disease risks for individuals show even greater promise of insights that could ultimately provide cost-effective and proactive healthcare with great efficacy.

## Data Availability

No datasets were generated or analyzed for this study.

## Author Contributions

DH conceived and wrote the manuscript. MW and RS advised DH and commented on the manuscript. WS and JO’S supervised DH and co-wrote the manuscript.

## Conflict of Interest Statement

The authors declare that the research was conducted in the absence of any commercial or financial relationships that could be construed as a potential conflict of interest.

## References

[B1] Abraham G. Inouye M. (2015). Genomic risk prediction of complex human disease and its clinical application. *Curr. Opin. Genet. Dev.* 33 10–16. 10.1016/j.gde.2015.06.005 26210231

[B2] Abraham G. Kowalczyk A. Zobel J. Inouye M. (2013). Performance and robustness of penalized and unpenalized methods for genetic prediction of complex human disease. *Genet. Epidemiol.* 37 184–195. 10.1002/gepi.21698 23203348

[B3] Abraham G. Tye-Din J. A. Bhalala O. G. Kowalczyk A. Zobel J. Inouye M. (2014). Accurate and robust genomic prediction of celiac disease using statistical learning. *PLoS Genet.* 10:e1004137. 10.1371/journal.pgen.1004137 24550740 PMC3923679

[B4] Alipanahi B. Delong A. Weirauch M. T. Frey B. J. (2015). Predicting the sequence specificities of DNA- and RNA-binding proteins by deep learning. *Nat. Biotechnol.* 33 831–838. 10.1038/nbt.3300 26213851

[B5] Amin N. Van Duijn C. M. Janssens A. C. J. W. (2009). Genetic scoring analysis: a way forward in genome wide association studies? *Eur. J. Epidemiol.* 24 585–587. 10.1007/s10654-009-9387-y 19728114 PMC2762531

[B6] Ardlie K. G. Deluca D. S. Segre A. V. Sullivan T. J. Young T. R. Gelfand E. T. (2015). The Genotype-Tissue Expression (GTEx) pilot analysis: multitissue gene regulation in humans. *Science* 348 648–660. 10.1126/science.1262110 25954001 PMC4547484

[B7] Ashley E. A. Butte A. J. Wheeler M. T. Chen R. Klein T. E. Dewey F. E. (2010). Clinical assessment incorporating a personal genome. *Lancet* 375 1525–1535. 10.1016/S0140-6736(10)60452-7 20435227 PMC2937184

[B8] Austin P. C. Tu J. V. Ho J. E. Levy D. Lee D. S. (2013). Using methods from the data-mining and machine-learning literature for disease classification and prediction: a case study examining classification of heart failure subtypes. *J. Clin. Epidemiol.* 66 398–407. 10.1016/j.jclinepi.2012.11.008 23384592 PMC4322906

[B9] Auton A. Abecasis G. R. Altshuler D. M. Durbin R. M. Bentley D. R. Chakravarti A. (2015). A global reference for human genetic variation. *Nature* 526 68–74. 10.1038/nature15393 26432245 PMC4750478

[B10] Belsky D. W. Moffitt T. E. Sugden K. Williams B. Houts R. McCarthy J. (2013). Development and evaluation of a genetic risk score for obesity. *Biodemogr. Soc. Biol.* 59 85–100. 10.1080/19485565.2013.774628 23701538 PMC3671353

[B11] Boulesteix A. L. Janitza S. Kruppa J. König I. R. (2012). Overview of random forest methodology and practical guidance with emphasis on computational biology and bioinformatics. *Wiley Interdiscip. Rev. Data Min. Knowl. Discov.* 2 493–507. 10.1002/widm.1072

[B12] Breiman L. E. O. (2001). Random forest. *Mach. Learn.* 45 5–32. 10.1023/A:1010933404324

[B13] Capriotti E. Calabrese R. Casadio R. (2006). Predicting the insurgence of human genetic diseases associated to single point protein mutations with support vector machines and evolutionary information. *Bioinformatics* 22 2729–2734. 10.1093/bioinformatics/btl423 16895930

[B14] Carayol J. Tores F. König I. R. Hager J. Ziegler A. (2010). Evaluating diagnostic accuracy of genetic profiles in affected offspring families. *Stat. Med.* 29 2359–2368. 10.1002/sim.4006 20623818 PMC2939926

[B15] Casson R. J. Farmer L. D. M. (2014). Understanding and checking the assumptions of linear regression: a primer for medical researchers. *Clin. Exp. Ophthalmol.* 42 590–596. 10.1111/ceo.12358 24801277

[B16] Che R. Motsinger-Reif A. (2013). Evaluation of genetic risk score models in the presence of interaction and linkage disequilibrium. *Front. Genet.* 4:138. 10.3389/fgene.2013.00138 23888168 PMC3719135

[B17] Chen X. Ishwaran H. (2012). Random forests for genomic data analysis. *Genomics* 99 323–329. 10.1016/j.ygeno.2012.04.003 22546560 PMC3387489

[B18] Clayton D. G. (2009). Prediction and interaction in complex disease genetics: experience in type 1 diabetes. *PLoS Genet.* 5:e1000540. 10.1371/journal.pgen.1000540 19584936 PMC2703795

[B19] Corinna C. Vladimir V. (1995). Support-vector networks. *Mach. Learn.* 20 273–297. 10.1007/BF00994018

[B20] Cox D. R. (1958). The regression analysis of binary sequences. *J. R. Stat. Soc.* 20 215–242. 10.1007/978-3-642-33442-9_35

[B21] Cruz J. A. Wishart D. S. (2006). Applications of machine learning in cancer prediction and prognosis. *Cancer Inform.* 2 59–77. 10.1177/117693510600200030 PMC2675494 19458758

[B22] Dai J. Y. LeBlanc M. Goodman P. J. Lucia M. S. Thompson I. M. Tangen C. M. (2018). Case-only methods identified genetic loci predicting a subgroup of men with reduced risk of high-grade prostate cancer by finasteride. *Cancer Prev. Res.* 12 113–120. 10.1158/1940-6207.CAPR-18-0284 30538099 PMC6365187

[B23] Dasgupta A. Sun Y. V. König I. R. Bailey-Wilson J. E. Malley J. D. (2011). Brief review of regression-based and machine learning methods in genetic epidemiology: the Genetic Analysis Workshop 17 experience. *Genet. Epidemiol.* 35 5–11. 10.1002/gepi.20642 22128059 PMC3345521

[B24] Dudbridge F. (2013). Power and predictive accuracy of polygenic risk scores. *PLoS Genet.* 9:e1003348. 10.1371/journal.pgen.1003348 23555274 PMC3605113

[B25] Escott-Price V. Sims R. Bannister C. Harold D. Vronskaya M. Majounie E. (2015). Common polygenic variation enhances risk prediction for Alzheimer’s disease. *Brain* 138 3673–3684. 10.1093/brain/awv268 26490334 PMC5006219

[B26] Evans D. M. Visscher P. M. Wray N. R. (2009). Harnessing the information contained within genome-wide association studies to improve individual prediction of complex disease risk. *Hum. Mol. Genet.* 18 3525–3531. 10.1093/hmg/ddp295 19553258

[B27] Fadason T. Ekblad C. Ingram J. R. Schierding W. S. Justin M. (2017). Physical interactions and expression quantitative traits loci identify regulatory connections for obesity and type 2 diabetes associated SNPs. *Front. Genet.* 8:150. 10.3389/fgene.2017.00150 29081791 PMC5645506

[B28] Fadason T. Schierding W. Lumley T. O’Sullivan J. M. (2018). Chromatin interactions and expression quantitative trait loci reveal genetic drivers of multimorbidities. *Nat. Commun.* 9:5198. 10.1038/s41467-018-07692-y 30518762 PMC6281603

[B29] Fourati S. Talla A. Mahmoudian M. Burkhart J. G. Klen R. Henao R. (2018). A crowdsourced analysis to identify ab initio molecular signatures predictive of susceptibility to viral infection. *Nat. Commun.* 9:4418. 10.1038/s41467-018-06735-8 30356117 PMC6200745

[B30] Furlong L. I. (2013). Human diseases through the lens of network biology. *Trends Genet.* 29 150–159. 10.1016/j.tig.2012.11.004 23219555

[B31] Geurts P. Irrthum A. Wehenkel L. (2009). Supervised learning with decision tree-based methods in computational and systems biology. *Mol. Biosyst.* 5 1593–1605. 10.1039/b907946g 20023720

[B32] Han J. (2018). “The design of diabetic retinopathy classifier based on parameter optimization SVM,” in *Proceedings of the 2018 International Conference Intelligence Informatics Biomedical Science*, (Shanghai), 52–58 10.1039/b907946g 20023720

[B33] He K. Zhang X. Ren S. Sun J. (2016). “Deep residual learning for image recognition,” in *Proceedings of the IEEE Conference Computer Vision Pattern Recognition*, (Silver Spring, MD), 770–778. 10.1109/ICIIBMS.2018.8549947

[B34] Hettige N. C. Cole C. B. Khalid S. De Luca V. (2016). Polygenic risk score prediction of antipsychotic dosage in schizophrenia. *Schizophr. Res.* 170 265–270. 10.1016/j.schres.2015.12.015 26778674

[B35] Huang Y. Wang P. (2012). Network based prediction model for genomics data analysis. *Stat. Biosci.* 4 1–23. 10.1007/s12561-012-9056-7 24348880 PMC3859188

[B36] Jiang R. Tang W. Wu X. Fu W. (2009). A random forest approach to the detection of epistatic interactions in case-control studies. *BMC Bioinformatics* 10:S65. 10.1186/1471-2105-10-S1-S65 19208169 PMC2648748

[B37] Johnson S. G. (2017). *Genomic Medicine in Primary Care,” in Genomic and Precision Medicine (Third Edition).* Amsterdam: Elsevier Inc., 1–18. 10.1186/1471-2105-10-S1-S65

[B38] Joseph P. V. Wang Y. Fourie N. H. Henderson W. A. (2018). A computational framework for predicting obesity risk based on optimizing and integrating genetic risk score and gene expression profiles. *PLoS One* 13:e0197843. 10.1371/journal.pone.0197843 29795655 PMC5993110

[B39] Jostins L. Barrett J. C. (2011). Genetic risk prediction in complex disease. *Hum. Mol. Genet.* 20 182–188. 10.1093/hmg/ddr378 21873261 PMC3179379

[B40] Kooperberg C. LeBlanc M. Obenchain V. (2010). Risk prediction using genome-wide association studies. *Genet. Epidemiol.* 34 643–652. 10.1002/gepi.20509 20842684 PMC2964405

[B41] Kruppa J. Ziegler A. König I. R. (2012). Risk estimation and risk prediction using machine-learning methods. *Hum. Genet.* 131 1639–1654. 10.1007/s00439-012-1194-y 22752090 PMC3432206

[B42] Kwak N. Choi C. H. (2002). Input feature selection for classification problems. *IEEE Trans. Neural Netw.* 13 143–159. 10.1109/72.977291 18244416

[B43] Laksman Z. Detsky A. S. (2011). Personalized medicine: understanding probabilities and managing expectations. *J. Gen. Intern. Med.* 26 204–206. 10.1007/s11606-010-1515-6 20878362 PMC3019312

[B44] Li Q. Diao S. Li H. He H. Li J. Y. (2018). Applying decision trees to establish risk rating model of breast cancer incidence based on non-genetic factors among Southwest China females. *Zhonghua Zhong Liu Za Zhi* 40 872–877. 10.3760/cma.j.issn.0253-3766.2018.11.015 30481942

[B45] López B. Torrent-Fontbona F. Viñas R. Fernández-Real J. M. (2017). Single nucleotide polymorphism relevance learning with random forests for type 2 diabetes risk prediction. *Artif. Intell. Med.* 85 43–49. 10.1016/j.artmed.2017.09.005 28943335

[B46] Lyall L. M. Wyse C. A. Morales C. A. C. Lyall D. M. Cullen B. Mackay D. (2018). Seasonality of depressive symptoms in women but not in men: a cross-sectional study in the UK Biobank cohort. *J. Affect. Disord.* 229 296–305. 10.1016/j.jad.2017.12.106 29329063

[B47] Manolio T. A. (2013). Bringing genome-wide association findings into clinical use. *Nat. Rev. Genet.* 14:549. 10.1038/nrg3523 23835440

[B48] Medicine G. Manolio T. A. (2013). Genomewide association studies and assessment of the risk of disease. *N. Engl. J. Med.* 363 166–176. 10.1038/nrg3523 20647212

[B49] Mehta P. Bukov M. Wang C.-H. Day A. G. R. Richardson C. Fisher C. K. (2019). A high-bias, low-variance introduction to Machine Learning for physicists. *Phys. Rep.* (in press). 10.1016/j.physrep.2019.03.001 PMC6688775 31404441

[B50] Mieth B. Kloft M. Rodríguez J. A. Sonnenburg S. Vobruba R. Morcillo-Suárez C. (2016). Combining multiple hypothesis testing with machine learning increases the statistical power of genome-wide association studies. *Sci. Rep.* 6 1–14. 10.1038/srep36671 27892471 PMC5125008

[B51] Mohri M. Rostamizadeh A. Talwalkar A. (2012). *Foundations of Machine Learning.* Cambridge, MA: MIT press. 10.1038/srep36671

[B52] Montañez C. A. C. Fergus P. Chalmers C. (2015). “Deep learning classification of polygenic obesity using genome wide association study SNPs,” in *Proceedings of the 2018 International Joint Conference on Neural Networks (IJCNN)*, (Budapest).

[B53] Nguyen T.-T. Huang J. Wu Q. Nguyen T. Li M. (2015). Genome-wide association data classification and SNPs selection using two-stage quality-based random forests. *BMC Genomics* 16:S5. 10.1186/1471-2164-16-S2-S5 25708662 PMC4331719

[B54] Nica A. C. Dermitzakis E. T. (2013). Expression quantitative trait loci: present and future. *Philos. Trans. Biol. Sci.* 368 1–6. 10.1098/rstb.2012.0362 23650636 PMC3682727

[B55] Niriella M. A. Kasturiratne A. Pathmeswaran A. De Silva S. T. Perera K. R. Subasinghe S. K. C. E. (2018). Lean non-alcoholic fatty liver disease (lean NAFLD): characteristics, metabolic outcomes and risk factors from a 7-year prospective, community cohort study from Sri Lanka. *Hepatol. Int.* 10.1007/s12072-018-9916-4 [Epub ahead of print]. 30539516

[B56] Nyaga D. M. Vickers M. H. Jefferies C. Perry J. K. O’Sullivan J. M. (2018). Type 1 diabetes mellitus-associated genetic variants contribute to overlapping immune regulatory networks. *Front Genet.* 9:535. 10.3389/fgene.2018.00535 30524468 PMC6258722

[B57] Okser S. Lehtimäki T. Elo L. L. Mononen N. Peltonen N. Kähönen M. (2010). Genetic variants and their interactions in the prediction of increased pre-clinical carotid atherosclerosis: the cardiovascular risk in young Finns study. *PLoS Genet.* 6:e1001146. 10.1371/journal.pgen.1001146 20941391 PMC2947986

[B58] Okser S. Pahikkala T. Airola A. Salakoski T. Ripatti S. Aittokallio T. (2014). Regularized machine learning in the genetic prediction of complex traits. *PLoS Genet.* 10:e1004754. 10.1371/journal.pgen.1004754 25393026 PMC4230844

[B59] Okser S. Pahikkala T. Aittokallio T. (2013). Genetic variants and their interactions in disease risk prediction - Machine learning and network perspectives. *BioData Min.* 6 1–16. 10.1186/1756-0381-6-5 23448398 PMC3606427

[B60] Ongen H. Brown A. A. Delaneau O. Panousis N. I. Nica A. C. Dermitzakis E. T. (2017). Estimating the causal tissues for complex traits and diseases. *Nat. Genet.* 49 1676–1683. 10.1038/ng.3981 29058715

[B61] Pahikkala T. Okser S. Airola A. Salakoski T. Aittokallio T. (2012). Wrapper-based selection of genetic features in genome-wide association studies through fast matrix operations. *Algorithms Mol. Biol.* 7 1–15. 10.1186/1748-7188-7-11 22551170 PMC3606421

[B62] Pal M. Foody G. M. (2010). Feature selection for classification of hyperspectral data by SVM. *IEEE Trans. Geosci. Remote Sens.* 48 2297–2307. 10.1109/TGRS.2009.2039484

[B63] Palaniappan S. Awang R. (2008). “Intelligent heart disease prediction system using data mining techniques,” in *Proceedings of the 2008 IEEE/ACS Int. Conf. Comput. Syst. Appl*, (Doha), 108–115. 10.1109/AICCSA.2008.4493524

[B64] Parker S. C. J. Stitzel M. L. Taylor D. L. Orozco J. M. Erdos M. R. Akiyama J. A. (2013). Chromatin stretch enhancer states drive cell-specific gene regulation and harbor human disease risk variants. *Proc. Natl. Acad. Sci. U.S.A.* 110 17921–17926. 10.1073/pnas.1317023110 24127591 PMC3816444

[B65] Purcell S. M. Wray N. R. Stone J. L. Visscher P. M. O’Donovan M. C. Sullivan P. F. (2009). Common polygenic variation contributes to risk of schizophrenia and bipolar disorder. *Nature* 460 748–752. 10.1038/nature08185 19571811 PMC3912837

[B66] Quinlan J. R. (1986). Induction of decision trees. *Mach. Learn.* 1 81–106. 10.1038/nature08185 19571811 PMC3912837

[B67] Quinlan J. R. (1990). Learning logical definitions from relations. *Mach. Learn.* 5 239–266. 10.1023/A:1022699322624

[B68] Rashkin S. R. Chua K. C. Ho C. Mulkey F. Jiang C. Mushiroda T. (2018). A pharmacogenetic prediction model of progression-free survival in breast cancer using genome-wide genotyping data from CALGB 40502 (Alliance). *Clin. Pharmacol. Ther.* 108 738–745. 10.1002/cpt.1241 30260474 PMC6379108

[B69] Redekop W. K. Mladsi D. (2013). The faces of personalized medicine: a framework for understanding its meaning and scope. *Value Heal.* 16 S4–S9. 10.1016/j.jval.2013.06.005 24034312

[B70] Ripatti S. Tikkanen E. Orho-Melander M. Havulinna A. S. Silander K. Sharma A. (2010). A multilocus genetic risk score for coronary heart disease: case-control and prospective cohort analyses. *Lancet* 376 1393–1400. 10.1016/S0140-6736(10)61267-6 20971364 PMC2965351

[B71] Rosenblatt F. (1962). *Principles of Neurodynamics: Perceptrons and the Theory of Brain Mechanisms. 1st Edition.* Ann Arbor, MI: Spartan Books, Michigan University 10.1016/S0140-6736(10)61267-6

[B72] Rusu V. Rusu V. Hoch E. Mercader J. M. Gymrek M. von Grotthuss M. (2017). Type 2 diabetes variants disrupt function of SLC16A11 through two distinct mechanisms. *Cell* 170 199–212.e20. 10.1016/j.cell.2017.06.011 28666119 PMC5562285

[B73] Salari K. Watkins H. Ashley E. A. (2012). Personalized medicine: hope or hype? *Eur. Heart J.* 33 1564–1570. 10.1093/eurheartj/ehs112 22659199 PMC3388016

[B74] Schaffer C. (1993). Technical note: selecting a classification method by cross-validation. *Mach. Learn.* 13 135–143. 10.1023/A:1022639714137

[B75] Schierding W. Antony J. Cutfield W. S. Horsfield J. A. O’Sullivan J. M. (2016). Intergenic GWAS SNPs are key components of the spatial and regulatory network for human growth. *Hum. Mol. Genet.* 25 3372–3382. 10.1093/hmg/ddw165 27288450

[B76] Schierding W. Antony J. Karhunen V. Vääräsmäki M. Franks S. Elliott P. (2018). GWAS on prolonged gestation (post-term birth): analysis of successive finnish birth cohorts. *J. Med. Genet.* 55 55–63. 10.1136/jmedgenet-2017-104880 29018042

[B77] Schierding W. O’Sullivan J. M. (2015). Connecting SNPs in diabetes: a spatial analysis of meta-GWAS loci. *Front. Endocrinol.* 6:102. 10.3389/fendo.2015.00102 26191039 PMC4490250

[B78] Schuster S. C. (2008). Next-generation sequencing transforms today’s biology. *Nat. Methods* 5 16–18. 10.1038/nmeth1156 18165802

[B79] Shi H. Kichaev G. Pasaniuc B. (2016). Contrasting the genetic architecture of 30 complex traits from summary association data. *Am. J. Hum. Genet.* 99 139–153. 10.1016/j.ajhg.2016.05.013 27346688 PMC5005444

[B80] Shieh Y. Eklund M. Madlensky L. Sawyer S. D. Thompson C. K. Stover Fiscalini A. (2017). Machine learning–based gene prioritization identifies novel candidate risk genes for inflammatory bowel disease. *Nat. Rev. Cancer* 12 1–12. 10.1016/j.tig.2017.09.004 28987266 PMC5701819

[B81] Shigemizu D. Abe T. Morizono T. Johnson T. A. Boroevich K. A. Hirakawa Y. (2014). The construction of risk prediction models using GWAS data and its application to a type 2 diabetes prospective cohort. *PLoS One* 9:e0092549. 10.1371/journal.pone.0092549 24651836 PMC3961382

[B82] Silver M. Chen P. Li R. Cheng C.-Y. Wong T.-Y. Tai E.-S. (2013). Pathways-driven sparse regression identifies pathways and genes associated with high-density lipoprotein cholesterol in two Asian cohorts. *PLoS Genet.* 9:e1003939. 10.1371/journal.pgen.1003939 24278029 PMC3836716

[B83] Singh G. Samavedham L. (2015). Unsupervised learning based feature extraction for differential diagnosis of neurodegenerative diseases: a case study on early-stage diagnosis of Parkinson disease. *J. Neurosci. Methods* 256 30–40. 10.1016/j.jneumeth.2015.08.011 26304693

[B84] So H. C. Sham P. C. Valencia A. (2017). Exploring the predictive power of polygenic scores derived from genome-wide association studies: a study of 10 complex traits. *Bioinformatics* 33 886–892. 10.1093/bioinformatics/btw745 28065900

[B85] Song J. Y. Perry A. M. Herrera A. F. Chen L. Skrabek P. Nasr M. (2018). New genomic model integrating clinical factors and gene mutations to predict overall survival in patients with diffuse large B-Cell lymphoma treated with R-CHOP. *Blood* 132(Suppl. 1):346. 10.1093/bioinformatics/btw745 28065900

[B86] Spiegel A. M. Hawkins M. (2012). “Personalized medicine” to identify genetic risks for type 2 diabetes and focus prevention: can it fulfill its promise? *Health Aff.* 31 43–49. 10.1377/hlthaff.2011.1054 22232093

[B87] The Wellcome Trust Case Control Consortium (2007). Genome-wide association study of 14 000 cases of seven common diseases and 3 000 shared controls. *Nature* 447 661–678. 10.1038/nature05911.Genome-wide 17554300 PMC2719288

[B88] Tibshirani R. (1996). Regression shrinkage and selection via the lasso. *J. R. Stat. Soc. Ser. B* 58 267–288. 10.1111/j.2517-6161.1996.tb02080.x

[B89] Touw W. G. Bayjanov J. R. Overmars L. Backus L. Boekhorst J. Wels M. (2013). Data mining in the life science swith random forest: a walk in the park or lost in the jungle? *Brief. Bioinform.* 14 315–326. 10.1093/bib/bbs034 22786785 PMC3659301

[B90] Vihinen M. (2012). How to evaluate performance of prediction methods? Measures and their interpretation in variation effect analysis. *BMC Genomics* 13(Suppl. 4):S2. 10.1186/1471-2164-13-S4-S2 22759650 PMC3303716

[B91] Vihinen M. (2013). Guidelines for reporting and using prediction tools for genetic variation analysis. *Hum. Mutat.* 34 275–277. 10.1002/humu.22253 23169447

[B92] Visscher P. M. Brown M. A. McCarthy M. I. Yang J. (2012). Five years of GWAS discovery. *Am. J. Hum. Genet.* 90 7–24. 10.1016/j.ajhg.2011.11.029 22243964 PMC3257326

[B93] Visscher P. M. Wray N. R. Zhang Q. Sklar P. McCarthy M. I. Brown M. A. (2017). 10 years of GWAS discovery: biology, function, and translation. *Am. J. Hum. Genet.* 101 5–22. 10.1016/j.ajhg.2017.06.005 28686856 PMC5501872

[B94] Wald N. J. Morris J. K. (2012). Personalized medicine: hope or hype. *Eur. Heart J.* 33 1553–1554. 10.1093/eurheartj/ehs089 22659200

[B95] Wang X. Strizich G. Hu Y. Wang T. Kaplan R. C. Qi Q. (2016). Genetic markers of type 2 diabetes: progress in genome-wide association studies and clinical application for risk prediction. *J. Diabetes* 8 24–35. 10.1111/1753-0407.12323 26119161

[B96] Wei L. Liao M. Gao Y. Ji R. He Z. Zou Q. (2014). Improved and promising identificationof human microRNAs by incorporatinga high-quality negative set. *IEEE/ACM Trans. Comput. Biol. Bioinform.* 11 192–201. 10.1109/TCBB.2013.146 26355518

[B97] Wei Z. Wang K. Qu H.-Q. Zhang H. Bradfield J. Kim C. (2009). From disease association to risk assessment: an optimistic view from genome-wide association studies on type 1 diabetes. *PLoS Genet.* 5:e1000678. 10.1371/journal.pgen.1000678 19816555 PMC2748686

[B98] Wei Z. Wang W. Bradfield J. Li J. Cardinale C. Frackelton E. (2013). Large sample size, wide variant spectrum, and advanced machine-learning technique boost risk prediction for inflammatory bowel disease. *Am. J. Hum. Genet.* 92 1008–1012. 10.1016/j.ajhg.2013.05.002 23731541 PMC3675261

[B99] Worachartcheewan A. Shoombuatong W. Pidetcha P. Nopnithipat W. Prachayasittikul V. Nantasenamat C. (2015). Predicting metabolic syndrome using the random forest method. *Sci. World J.* 2015 1–10. 10.1155/2015/581501 26290899 PMC4531182

[B100] Wray N. Goddard M. Visscher P. (2007). Prediction of individual genetic risk to disease from genome-wide association studies. *Genome Res.* 17 1520–1528. 10.1101/gr.6665407.1520 17785532 PMC1987352

[B101] Wray N. R. Lee S. H. Mehta D. Vinkhuyzen A. A. E. Dudbridge F. Middeldorp C. M. (2014). Research review: polygenic methods and their application to psychiatric traits. *J. Child Psychol. Psychiatry Allied Discip.* 55 1068–1087. 10.1111/jcpp.12295 25132410

[B102] Wu T. T. Chen Y. F. Hastie T. Sobel E. Lange K. (2009). Genome-wide association analysis by lasso penalized logistic regression. *Bioinformatics* 25 714–721. 10.1093/bioinformatics/btp041 19176549 PMC2732298

[B103] Xue L. Tang B. Chen W. Luo J. (2018). Prediction of CRISPR sgRNA activity using a deep convolutional neural network. *J. Chem. Inf. Model.* 59 615–624. 10.1021/acs.jcim.8b00368 30485088

[B104] Young T. Hazarika D. Poria S. Cambria E. (2018). Recent trends in deep learning based natural language processing [Review Article]. *IEEE Comput. Intell. Mag.* 13 55–75. 10.1109/MCI.2018.2840738 28410513

[B105] Yu F. Rybar M. Uhler C. Fienberg S. E. (2014). “Differentially-private logistic regression for detecting multiple-SNP association in GWAS databases,” in *Privacy in Statistical Databases*, ed. Domingo-Ferrer J. (Cham: Springer International Publishing), 170–184.

[B106] Yu W. (2010). Application of support vector machine modeling for prediction of common diseases: the case of diabetes and pre-diabetes. *BMC Med. Inform. Decis. Mak.* 10:16. 10.1186/1472-6947-10-16 20307319 PMC2850872

[B107] Yuan Y. (2008). Step-sizes for the gradient method. *AMS IP Stud. Adv. Math.* 42:785.

[B108] Zhang D. Shen D. (2012). Multi-modal multi-task learning for joint prediction of multiple regression and classification variables in Alzheimer’s disease. *Neuroimage* 59 895–907. 10.1016/j.neuroimage.2011.09.069 21992749 PMC3230721

[B109] Zhang Z. Zhao Y. Liao X. Shi W. Li K. Zou Q. (2018). Deep learning in omics: a survey and guideline. *Brief. Funct. Genomics* 10.1093/bfgp/ely030 [Epub ahead of print]. 30265280

[B110] Zhou J. Troyanskaya O. G. (2015). Predicting effects of noncoding variants with deep learning-based sequence model. *Nat. Methods* 12 931–934. 10.1038/nmeth.3547 26301843 PMC4768299

[B111] Zou H. Hastie T. (2005). Regularization and variable selection via the elastic net. *J. R. Stat. Soc. Ser. B* 67 301–320. 10.1111/j.1467-9868.2005.00503.x

